# High-Density Linkage Maps Based on Genotyping-by-Sequencing (GBS) Confirm a Chromosome-Level Genome Assembly and Reveal Variation in Recombination Rate for the Pacific Oyster *Crassostrea gigas*

**DOI:** 10.1534/g3.120.401728

**Published:** 2020-11-03

**Authors:** Xiaoshen Yin, Alberto Arias-Pérez, Tevfik Hamdi Kitapci, Dennis Hedgecock

**Affiliations:** Department of Biological Sciences, University of Southern California, Los Angeles, California, 90089

**Keywords:** linkage mapping, genotyping-by-sequencing, genome assembly, recombination rate, Pacific oyster *Crassostrea gigas*

## Abstract

Studies of linkage and linkage mapping have advanced genetic and biological knowledge for over 100 years. In addition to their growing role, today, in mapping phenotypes to genotypes, dense linkage maps can help to validate genome assemblies. Previously, we showed that 40% of scaffolds in the first genome assembly for the Pacific oyster *Crassostrea gigas* were chimeric, containing single nucleotide polymorphisms (SNPs) mapping to different linkage groups. Here, we merge 14 linkage maps constructed of SNPs generated from genotyping-by-sequencing (GBS) methods with five, previously constructed linkage maps, to create a compendium of nearly 69 thousand SNPs mapped with high confidence. We use this compendium to assess a recently available, chromosome-level assembly of the *C. gigas* genome, mapping SNPs in 275 of 301 contigs and comparing the ordering of these contigs, by linkage, to their assembly by Hi-C sequencing methods. We find that, while 26% of contigs contain chimeric blocks of SNPs, *i.e.*, adjacent SNPs mapping to different linkage groups than the majority of SNPs in their contig, these apparent misassemblies amount to only 0.08% of the genome sequence. Furthermore, nearly 90% of 275 contigs mapped by linkage and sequencing are assembled identically; inconsistencies between the two assemblies for the remaining 10% of contigs appear to result from insufficient linkage information. Thus, our compilation of linkage maps strongly supports this chromosome-level assembly of the oyster genome. Finally, we use this assembly to estimate, for the first time in a Lophotrochozoan, genome-wide recombination rates and causes of variation in this fundamental process.

The physical, linear arrangement of genes in DNA molecules has profound implications at all levels of biological organization, from cell, to organism, to population. Thus, studies of linkage and linkage mapping have been fundamental for advancing genetic and biological knowledge for over 100 years (Morgan 1911; Sturtevant 1913). Today, linkage maps are essential for mapping phenotypes to genotypes, whether through quantitative-trait loci (QTL) mapping, genome-wide association studies (GWAS), or genomic selection (GS). A further, important use of dense linkage maps, however, is the validation of genome assemblies (Lewin *et al.* 2009; Dalloul *et al.* 2010; Dodgson *et al.* 2011; Fierst 2015; Hedgecock *et al.* 2015; Verde *et al.* 2017).

The Pacific oyster *Crassostrea gigas* is a species of global commercial value and scientific interest, having been introduced from Asia to all continents but Antarctica for aquaculture (Mann 1979). Oyster cytogenetics has been fairly well studied. Cupped oysters of the genus *Crassostrea* have 10 pairs of chromosomes (Ahmed and Sparks 1967; Longwell *et al.* 1967); in the Pacific oyster, chromosomes are metacentric or sub-metacentric (Thiriot-Quievreux 1984). Longwell *et al.* (1967, their Figure 1) show 10 pairs of diakinesis chromosomes in an unfertilized egg of the eastern oyster *Crassostrea virginica*; eight chromosomes have a single crossover and two chromosomes have two crossovers, a total of 12 crossovers, for a cytological estimate of 600 cM map-length (1.2 crossovers per bivalent × 50 cM × 10 chromosomes). Li and Guo (2004) subsequently reported averages of 1.1 to 1.2 chiasmata per chromosome in the Pacific oyster. These cytological estimates of map length correspond well with the genome size of 559 Mb subsequently provided by Zhang *et al.* (2012).

**Figure 1:**
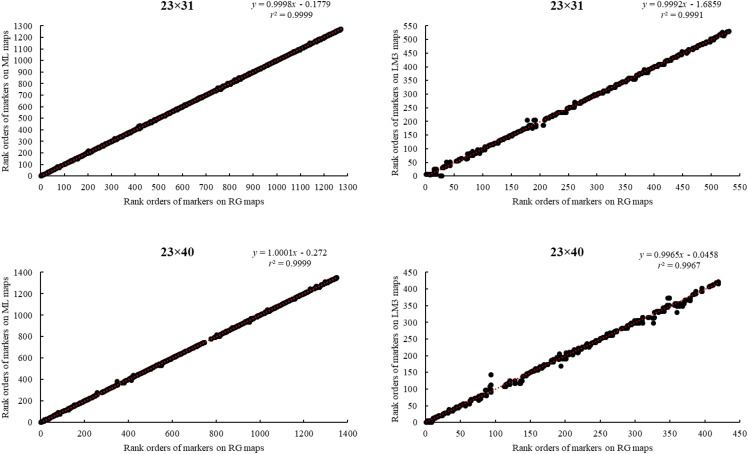

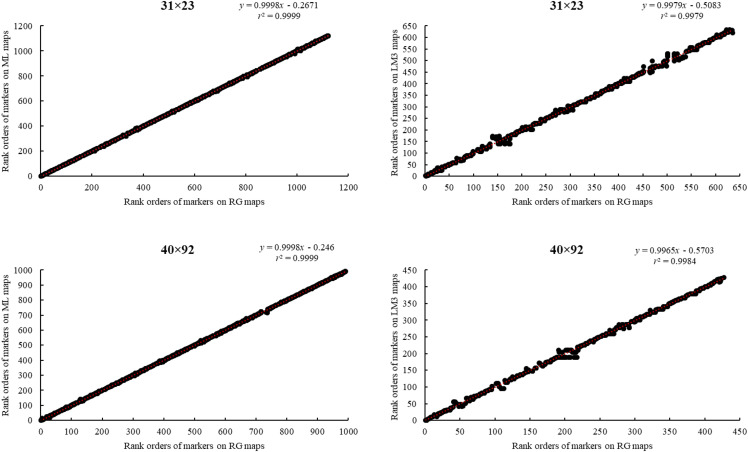

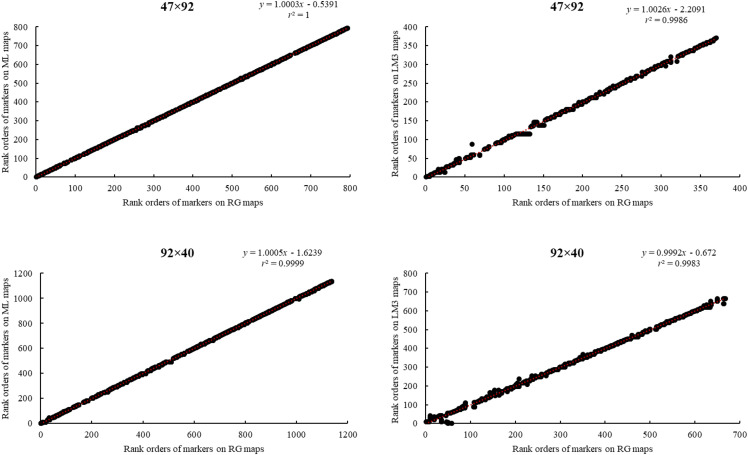
Correlations among rank orders of common markers on maps made using the regression (RG) and maximum likelihood (ML) methods of JoinMap 4.1 and Lep-MAP3 (LM3).

Allozymes furnished the first evidence of genetic linkage in bivalve molluscs and the first partial linkage or gene-centromere maps (Foltz 1986; Guo and Gaffney 1993; Beaumont 1994; McGoldrick and Hedgecock 1997). The development of DNA markers, thereafter, enabled construction of first-generation linkage maps for all ten linkage groups, based on AFLP markers (Li and Guo 2004), microsatellite DNA markers (Hubert and Hedgecock 2004; Li and Kijima 2006; Hubert *et al.* 2009; Plough and Hedgecock 2011), combinations of AFLP and microsatellite DNA markers (Guo *et al.* 2012) or of microsatellite DNA markers and single-nucleotide polymorphisms (SNPs; Sauvage *et al.* 2010; Zhong *et al.* 2014). These first-generation linkage maps had hundreds of markers and an average marker spacing of 8-10 cM, providing only limited resolution for mapping phenotypes to genotypes.

To increase map resolution and reproducibility, Hedgecock *et al.* (2015) constructed second-generation linkage maps from five families, using more than 1100 coding SNPs, together with microsatellites for alignment with the first-generation maps. Average marker spacing for these second-generation maps was about 1 cM, an almost 10-fold improvement in density over the first-generation linkage maps. On these maps, variation in marker orders and map distances among families and mapping methods were attributed to markers segregating from only one parent, widespread distortions of segregation ratios caused by early mortality, which had previously been observed (Bierne *et al.* 1998; Launey and Hedgecock 2001; Plough and Hedgecock 2011; Plough *et al.* 2016), and genotyping errors. More importantly, these second-generation linkage maps suggested widespread errors in the scaffold assemblies of the *C. gigas* genome (Zhang *et al.* 2012; GenBank assembly accession: GCA_000297895.1; hereafter, the v9 genome assembly), posing a significant impediment to locating candidate genes under QTL peaks (Hedgecock *et al.* 2015; Yin 2018).

Following advances in high-throughput sequencing technology, direct genotyping-by-sequencing (GBS) methods enabled the generation of a large number of genetic markers for non-model organisms (Elshire *et al.* 2011; Narum *et al.* 2013; Robledo *et al.* 2018), facilitating the construction, in principle, of higher-density linkage maps for non-traditional model species, such as the Pacific oyster. Here, we use GBS methods to create a set of reliable, higher-density, linkage maps, laying the groundwork for higher-resolution QTL mapping and the detection of genetic mechanisms underlying variation in viability, growth and sex determination. We also seek to validate the second of two, recently released, chromosome-level assemblies of the *C. gigas* genome (The Roslin Institute, February 19, 2020, GenBank assembly accession: GCA_902806645.1; The Institute of Oceanology, Chinese Academy of Sciences, February 27, 2020, GenBank assembly accession: GCA_011032805.1). The latter assembly (ASM1103280v1; hereafter, the “Chr_v1” genome assembly) was accomplished, using third-generation DNA sequencing methods and Hi-C analysis, to associate 301 contigs with an N50 of nearly 3.2 Mb (Qi *et al.* 2020).

The linkage maps constructed with GBS methods average less than 1 cM between markers, for seven F_2_ families and one, outcrossed, full-sib family. We use two different linkage-mapping strategies, one based on detailed processing of sequence data to call genotypes, followed by iterative mapping procedures, using regression and maximum likelihood methods in JoinMap 4.1 (Van Ooijen 2011), and the other based on genotype likelihoods calculated directly from variant call format (vcf) files or binary alignment (bam) files and one-step determination of linkage by Lep-MAP3 (Rastas 2017). As in our previous work (Hedgecock *et al.* 2015), we find that the use of multiple families, some related by descent, lends confidence in the statistical construction of linkage maps. We merge data on mapped SNPs from these third-generation linkage maps with data on mapped SNPs from the five, second-generation linkage maps (Hedgecock *et al.* 2015), compiling a compendium of 71,156 mapped SNPs, of which nearly 69 thousand are supported by information from more than one mapping family. We use this compendium to examine evidence for contig misassembly in the Chr_v1 assembly (Qi *et al.* 2020). We then use ALLMAPS (Tang *et al.* 2015) to assemble these contigs into a chromosome-level genome and compare this linkage-based assembly to the sequence-based, Chr_v1 assembly.

Understanding of recombination and variation in recombination across the genome is of fundamental interest in the evolution of eukaryotes (Nachman 2002; Stapley *et al.* 2017; Peñalba and Wolf 2020). One approach is to study patterns of linkage disequilibrium across species, which generates an indirect, long-term picture of patterns in recombination rates across genomes. Another approach is to look directly at genetic recombination in meiosis, using linkage mapping, which generates a snapshot of standing variation in recombination rates within and among individuals. Dense linkage maps and chromosome-level genome assemblies enable construction of recombination-rate (RR) profiles across chromosomes and exploration of factors affecting variation in RR within and among individuals (Yu *et al.* 2001; Kong *et al.* 2002; Myers *et al.* 2005; Chan *et al.* 2012; Dukić *et al.* 2016; Gion *et al.* 2016).

Here, we use high-density linkage maps for six, interrelated F_2_ families—2,082 meioses in all, from 12 parents—combined with the chromosome-level assembly of the oyster genome, to estimate, for the first time in a Lophotrochozoan, a genome-wide recombination rate. We obtain profiles of RR within chromosomes and examine sources of variation in RR among families, between the sexes of parents, and among chromosomes.

## Materials and Methods

### Mapping families

Our study was based on 12 families (Table S1)—F12, F20, F45, 2×10, 51×35, 23×31, 23×40, 31×23, 40×92, 47×92, 92×40, and 58×19 (sire × dam). Hedgecock *et al.* (2015) set up families F12, F20, F45, 2×10 and 51×35. We derived six, interrelated F_2_ families, 23×31, 23×40, 31×23, 40×92, 47×92 and 92×40, from crosses of full-sib F_1_ hybrids, which were, in turn, produced by crosses of partially inbred lines (23, 31, 40, 47 and 92) in 2009. G_0_ families 23, 31, 47 and 92 were established using wild-caught parents by the Molluscan Broodstock Program (MBP) in 1996 (Langdon *et al.* 2003). G_0_ family 40 was established using wild-caught parents at the Taylor Shellfish Farms hatchery in 2001. After one or two generations of inbreeding, five partially inbred lines, 23, 31, 40, 47 and 92, were among seven parent lines used for a diallel cross at the Taylor hatchery in 2009. In May 2011, adults from families produced by this diallel cross became parents of F_2_ families through brother-sister crossing. We reared F_2_ families in Thorndyke Bay, WA, and sampled them in October 2012. Family 58×19 was made from a controlled pair-cross of wild-caught parents collected from Pipestem Inlet, Vancouver Island, British Columbia, Canada and used in a study of juvenile oyster mortality caused by the OsHV-1 virus in Tomales Bay, CA in 2015 (Kitapci *et al.* 2018; Kitapci 2018).

### Genotyping-by-sequencing (GBS), GATK analysis, SNP discovery, and parentage analysis

Except for the families genotyped by Hedgecock *et al.* (2015), all other families were harvested and shipped to the University of Southern California, where adductor muscle tissue was dissected and preserved in 70% ethanol for later DNA extraction. We obtained genotype data for the F_2_ families and for family 58×19, using GBS, and the follow-up bioinformatics analyses were guided by Genome Analysis Toolkit (GATK, https://www.broadinstitute.org/gatk/) (Bentley *et al.* 2008; McKenna *et al.* 2010; Elshire *et al.* 2011). We re-genotyped family 51×35 because we used this family to develop the GBS-GATK genotyping methods for this study.

GBS involved two steps, library preparation and sequencing. To construct libraries for sequencing, we first extracted DNA from all parents and progeny, following the DNeasy 96 Procotol, Purification of Total DNA from Animal Tissues (Qiagen, https://www.qiagen.com/us/shop/sample-technologies/dna/genomic-dna/dneasy-blood-and-tissue-kit/#resources), with minor modifications. We checked the quality of the extracted DNA by agarose gel electrophoresis, quantified DNA in each sample, following the protocol of Quant-iT PicoGreen dsDNA Assay Kit, and diluted or concentrated the sample DNA to a working concentration of 10 ng/µl. To make libraries for sequencing, we digested 100 ng of extracted genomic DNA in a final volume of 20 μL with the restriction enzyme *Apo*I (NEB # R0566L), in the buffer supplied by the manufacturer, at 50° for 2 h, and then at 80° for 20 min. We ligated common and barcoded adapters (designed with http://www.deenabio.com/services/gbs-adapters) to genomic DNA by incubating the mixture of digested genomic DNA, T4 DNA ligase (NEB # M0202L), H_2_O, and 10× T4 DNA ligase reaction buffer at 22° for 60 min, 65° for 30 min, and 4° for cooling (Table S2). We then pooled and cleaned the ligated products from different samples. We amplified the pooled products in 2 µl DNA template, 21 µl H_2_O, 25 µl NEB 2× Taq Master mix (NEB # M0270S), and 2 µl Primer mix, using the PCR program, 5 min at 72°, 30 s at 98°, 14 cycles × (10 s at 98°, 30 s at 65°, 30 s at 72°), 5 min at 72°, and holding at 4° (Table S2). We then purified the amplified products by agarose gel electrophoresis, following protocols of QIAquick PCR Purification Kit, and extracted the DNA fragments, following the protocol of MinElute Gel Extraction Kit. Once libraries were constructed, we checked their size distributions on an Agilent Bioanalyzer and quantified their concentration using NanoDrop 2000 spectrophotometer. Sequencing libraries comprised 96 bar-coded samples, except for family 23×31, which was sequenced in libraries containing 48 samples; this resulted in about 2× greater sequencing depth and SNPs in family 23×31 than in the other families (Table S3).

We sent all libraries to the University of Southern California (USC) Genome & Cytometry Core for sequencing and sequenced each library in a single lane on an Illumina HiSeq instrument. We processed the GBS data generated from sequencing on the Linux clusters of the University of Southern California’s Center for High-Performance Computing. Sequences that matched a barcode (one mismatch allowed), followed by the *Apo*I remnant site (one mismatch allowed), were assigned to the corresponding sample using a custom script in Python. The script also truncated reads having a full cut site or the beginning of the common adapter.

For the six, interrelated F_2_ families, we aligned reads to the then-available v9 genome assembly, using the Burrows-Wheeler alignment tool (BWA v0.7.8, MEM algorithm), and processed alignments with the GATK software package v3.3.0 (McKenna *et al.* 2010) for local realignment around indels and base quality score recalibration. Variant and genotype calling were done with the GATK HaplotypeCaller tool and refined by variant quality score recalibration with the same software (Van der Auwera *et al.* 2013). To carry out this process, a training set was prepared, using replicated individuals. Data from those individuals were filtered for the number of reads (minimum of 15), genotyping quality (minimum of 20), and sites, for which replicates had the same genotype and allelic balance was between 0.35 and 0.65. After processing data with GATK, we filtered the results using VCFtools v0.1.12b. We removed indels and kept biallelic sites with genotypes having a minimum genotyping quality of 30 and a minimum of five reads per site per individual. We excluded sites genotyped for less than 80% of individuals and individuals with data for less than 70% of sites. After excluding sites with missing data for parents, sites that were homozygous in both parents, and sites at which offspring had monomorphic or unexpected genotypes, we coded the genotypes of the remaining sites into the format required by JoinMap 4.1. We named each SNP by its location on the v9 genome assembly (*i.e.*, scaffold number and nucleotide position). The pedigrees of all well-genotyped progeny for the six, interrelated F_2_ families (n = 1,166) were confirmed by relatedness analysis (Manichaikul *et al.* 2010) and CERVUS (Marshall *et al.* 1998). Individuals that did not match their parents or siblings (n = 125) were removed.

### Linkage analysis using JoinMap 4.1 (JM)

For family 51×35 and the six, interrelated F_2_ families, we conducted linkage analyses with JoinMap 4.1 (Van Ooijen 2011), using the cross-pollinated (CP) coding of genotypes. We observed three mating types in the F_2_ families, hk×hk, lm×ll and nn×np, among which hk×hk represented bi-parentally segregating markers, while lm×ll and nn×np represented maternally and paternally segregating markers, respectively (note that JoinMap 4.1 notation is dam×sire). To correct genotyping errors and fill missing genotype information, we first imputed uni-parentally segregating markers using the program Maskov according to Ward *et al.* (2013). Then, we pooled imputed, uni-parentally and raw, bi-parentally segregating markers together for linkage mapping. We excluded loci with fewer than 10% of individuals genotyped per family. To increase the efficiency of linkage mapping, we retained only one marker from each set of identical markers (“identicals,” *i.e.*, a group of markers assigned to the same position on linkage maps) identified by JoinMap 4.1. Markers were grouped, using the independence LOD, which ranged from 2 to 26, across linkage groups and families.

We used both regression (RG) and maximum likelihood (ML) mapping methods to construct linkage maps. For the RG method, we used the Kosambi mapping function, with maximum recombination frequency of 0.4, minimum LOD of 1.0, and goodness-of-fit jump threshold for removing loci of 5.0. All other parameters were set to default values. We used only the first- or second-round regression linkage maps. For the ML method, which uses Haldane mapping units, we set chain length to 5,000, length of burn-in chain to 20,000, number of Monte Carlo EM cycles to 10, and chain length per Monte Carlo EM cycle to 5,000. All other parameters were set to default values. The parameter used to determine whether a locus fits well between its neighboring loci is nearest-neighbor fit (cM), with a larger value indicating a poor fit (Van Ooijen 2011). We constructed a map for each linkage group, using the RG method. If the largest nearest-neighbor fit was greater than 5 cM for any marker, we excluded that marker and constructed another RG map. We repeated this process until the largest nearest-neighbor fit was smaller than 5 cM and termed the result the initial RG map. Using the markers on this initial RG map, we then constructed a ML map. If nearest-neighbor fit for any marker was larger than 5 cM, we removed that marker and reconstructed both RG and ML maps. We repeated this iterative process until the largest nearest-neighbor fits were less than 5 cM for RG and ML maps. We evaluated the consistency between RG and ML maps by *r^2^*, the correlation coefficient for linear regression of marker rank orders on the ML map against marker rank orders on the RG map. When *r^2^* was less than 0.95, we removed markers with large nearest-neighbor fits or markers with inconsistent positions between RG and ML maps. We considered RG and ML maps for a linkage group consistent when *r^2^* reached at least 0.95; we then took the RG map as the final linkage map for that linkage group and brought back identical markers if their representative remained on the final RG map. Linkage groups were numbered according to the second-generation linkage map in Hedgecock *et al.* (2015), by matching the scaffold numbers of markers on our final linkage maps with those on the maps constructed by Hedgecock *et al.* (2015).

### Linkage analysis using Lep-MAP3 (LM3)

For the six, interrelated F_2_ families and family 58×19, we first mapped trimmed reads using BWA with default parameters (Li and Durbin 2009) to the v9 genome assembly (Zhang *et al.* 2012), and generated a pileup file using “mpileup” command (parameters “-q 10 -Q 10 -s”) with SAMTOOLS (version 1.5) (Li *et al.* 2009). Then, we estimated genotype likelihoods from this mpileup file and constructed a linkage map using LM3 (Rastas 2017). We called module ParentCall2 with parameter removeNonInformative = 1, module Filtering2 with default parameters, and module SeparateChromosomes2 to group markers. The parameter lodLimit of module SeparateChromosomes2 is crucial in map construction, so we optimized it with two criteria: first, ∼90% of markers are on the first ten linkage groups; second, markers are distributed close to uniformly on these ten linkage groups. Optimized lodLimits ranged from 22 to 43. We next called module JoinSingles2All with default parameters. Finally, we called module OrderMarkers2 to order markers within each linkage group with parameters outputPhasedData = 1 and sexAveraged = 1. We only considered positions that were heterozygous in both parents. We retained one marker of each identical group for linkage analysis, using LM3, but brought back identical markers if their representative remained on the final LM3 map.

### Linkage map comparison and mapping method assessment

We first estimated genome coverage, according to (1) (Bishop *et al*. 1983) for all newly constructed linkage maps,

$$\text{GC}=1-e^{-2*dn/L},$$ (1)

where *d* is the average distance between neighboring markers and *n* is the total number of markers on ten linkage groups in each family; the total length of a linkage group is estimated by adding twice the average distance between neighboring markers to the map length of the corresponding linkage group; *L* is sum of total lengths of ten linkage groups in a family. Since we conducted linkage analysis using both mapping methods for the six, interrelated F_2_ families, we compared map lengths, numbers of markers (without identicals), average spacing (between unique mapping positions), and genome coverage between the JM and LM3 maps of these six families, using Student’s *t*-tests (*i.e.*, testing means of paired two samples). We evaluated the reliability and consistency of different linkage mapping methods, by comparing rank orders of common markers on linkage maps constructed using RG method in JM, ML method in JM, and LM3 for the six, interrelated F_2_ families (Figure 1).

### Linkage-based assembly of the Pacific oyster Crassostrea gigas genome

After release of the Chr_v1 assembly (Qi *et al.* 2020), we remapped all SNPs from 19 linkage maps to this genome. The correspondence between the linkage group numbering of Hedgecock *et al.* (2015) and the chromosome numbering of the Chr_v1 genome assembly is in Table S4. To remap SNPs on the v9 genome assembly to the Chr_v1 genome assembly, we first remapped all SNPs to the contigs in the Chr_v1 genome assembly. We extracted reads overlapping with SNPs from sequences for families F12, F20, F45, 2×10, and 51×35, from the ten largest BAM files for families 23×31, 23×40, 31×23, 40×92, 47×92, and 92×40, and from the five largest BAM files for family 58×19. We aligned these reads to the contigs with BWA and then realigned them using the tool IndelRealigner provided by GATK (McKenna *et al.* 2010). We removed alignments with low mapping quality (< 20), tagged with secondary or supplementary alignments, or with a high percentage of soft clipped bases (> 50%). We computed the position of SNPs in the contigs, using the CIGAR string and in-house python scripts. For most SNPs, all or most of the reads aligned to a unique position. We discarded a marker when a notable number of reads (>10%) pointed to a different position. If different SNPs on the v9 genome assembly were translated to the same position on the contigs, we removed these SNPs. In total, 71,220 of 97,987 SNPs were kept after remapping from the v9 genome assembly to the Chr_v1 contigs; these were renamed with a Chr_v1 chromosome number and nucleotide position. Excluded from further analyses were 64 SNPs that mapped to either two or three different linkage groups. With the remaining 71,156 SNPs, we created a compendium based on 12 families (Table S5).

We identified the linkage group, to which the largest number of SNPs on a contig were assigned, as the consensus linkage group of that contig. If a SNP was assigned to the consensus linkage group, this SNP was taken as being correctly grouped by linkage mapping. We constructed a three-way, loglinear model to test whether JM or LM3 grouped SNPs more accurately, whether correctly grouped markers were more likely to be supported by linkage information from more than one family, and whether mapping method and level of family support were independent.

**Figure 2:**
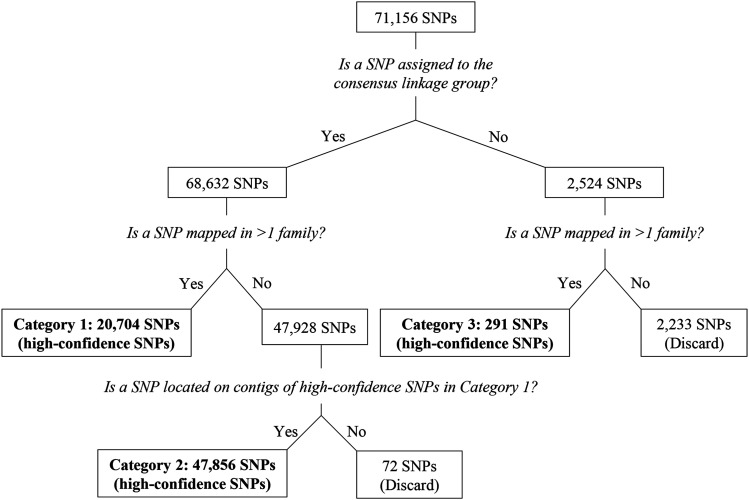
Decision tree for selecting high-confidence SNPs.

To assemble a *C. gigas* genome based on linkage information and given the potential for a given SNP to be inaccurately grouped, we used a decision tree (Figure 2) to identify SNPs mapped with high-confidence. Briefly, a SNP was defined as mapped with high-confidence, if it fell into any of the three categories: (1) assigned to the consensus linkage group and mapped in more than one family, (2) assigned to the consensus linkage group, mapped in one family but located on contigs with SNPs in category (1), or (3) assigned to a non-consensus linkage group in more than one family.

We input all high-confidence SNPs on 19 linkage maps from 12 families to ALLMAPS, to order and orient the Chr_v1 contigs (Tang *et al.* 2015). By merging all 19 linkage maps, ALLMAPS first generated two files, a bed file and a weights file, in which the weight of each map was set to 1. Then, using the bed file and the Chr_v1 contig fasta files, ALLMAPS ordered and oriented contigs to generate an assembly of all contigs containing high-confidence SNPs demonstrated in a fasta file.

By checking whether there is a block of contiguous SNPs on a contig assigned to linkage groups different than the consensus linkage group of the contig (*i.e.*, a chimeric block), we evaluated the Chr_v1 genome assembly at the contig level. By comparing the ordering and orientation of the Chr_v1 contigs with that on the ALLMAPS-generated assembly, we assessed the Chr_v1 genome assembly at the chromosome level.

### Recombination rate

To assess variation in recombination rate (RR), we used the ML maps for each parent of the six, interrelated F_2_ families, which enabled analyses of variation in RR among family, sex and chromosome. The ML method provides recombination-frequency maps for each parent, although the genetic distances appear to be overestimated, in part because they assume no crossover interference. We first divided the genetic distance (in cM) between the two most distal SNPs on each linkage group by the physical distance between these SNPs on the corresponding chromosome of the Chr_v1 genome assembly. Next, we conducted a three-way ANOVA with family, sex, chromosome and two-way interactions among them as independent variables and RR as the dependent variable, to test whether these factors make a significant contribution to variation in recombination rate. Since we do not have replication of each family-by-sex-by-chromosome combination, the significance of the three-way interaction among family, sex and chromosome cannot be estimated and is thus excluded from ANOVA.

To assess variation in recombination rate along each chromosome, we calculated recombination rates across the ten chromosomes of the six, interrelated F_2_ families using MareyMap (Rezvoy *et al.* 2007) in R 3.6.1. We used genetic positions of SNPs on the RG (*i.e.*, RG-based recombination rate) and ML (*i.e.*, ML-based recombination rate) maps and physical positions of SNPs on the Chr_v1 genome assembly. We first removed outlier loci whose genetic distances did not increase monotonically with their physical positions, as defined by the instruction on MareyMap, because these outlier loci could arise from mapping errors on genetic or physical maps. We used the loess-based method, setting Span to 0.3 and Degree to 1. We removed loci with negative recombination rates and calculated recombination rates for the remaining set of loci. After standardizing RR values from ML maps to facilitate comparisons among families, we tested the null hypothesis that values below -1.28 and above 1.28, nominal 10^th^ and 90^th^ percentiles, were randomly distributed across the chromosome, using Pearson’s chi-square test for complete spatial randomness as implemented by PROC SPP in SAS 9.4 (SAS Institute, Cary, NC). Analyses were done by family and by chromosome, using areas tightly defined by the length of chromosome mapped and the range in standardized RR values, nine quadrats per area, and a minimum of nine observations, so that the expected value in each quadrat was at least 1.0. We adjusted the probability threshold for significance at the α=5% level to 0.001 to account for multiple testing.

Lastly, in order to check whether there is reduced recombination around centromeres, we located the leftmost position of centromere-associated microsatellite markers detected by Hubert *et al.* (2009) on the Chr_v1 genome assembly, using the Burrows-Wheeler alignment tool (BWA v0.7.17, MEM algorithm). We mapped six markers unambiguously to the assembly (*uscCg205*, AY999703, on chr 2; *ucdCg147*, AF468549, on chr 3; *ucdCg028*, AF051178, on chr 5; *ucdCg197*, AF468595, also on chr 5; *imbCg44*, Y12085, on chr 7; and *imbCg049*, Y12086, on chr 8). Using the confidence limits for the gene-centromere distances from Hubert *et al.* (2009, Table 4), we identified the lowest RR in putative regions containing the centromere.

### Data availability

Supplemental material available at figshare: https://doi.org/10.25387/g3.13077470.

## Results

### Numbers of SNPs generated and mapped

In total, we obtained genotypes for 12 parent oysters and 1,041 progeny (Table S1) for the six, interrelated F_2_ families. By comparing the genotype at common loci between duplicates of parents of the six, interrelated F_2_ families, we found that genotyping error rate was 2–3%. For the six, interrelated F_2_ families, numbers of SNPs generated by GBS, which passed filtering and genetic criteria, range from 4,615 to 13,885 (Table S3). On average, 65% of these markers (ranging from 3,374 to 7,982) were input to JoinMap 4.1 (JM), of which nearly a third (32.5%) had identical genotypes. The number of markers placed on final RG maps ranges from 794 to 1,351 or from 16 to 34% of the markers input to JoinMap 4.1 for each family (Table S3).

### Linkage map comparison and mapping method assessment

Lengths of JM maps for the six, interrelated F_2_ families range from 454.6 cM to 589.7 cM, with average marker spacings from 0.445 cM to 0.856 cM (Table 1). Lengths of LM3 maps for these same families range from 585.8 cM to 943 cM, with average marker spacings from 0.483 cM to 0.714 cM (Table 1). Compared to their JM counterparts, LM3 maps have a larger length (*P* = 0.024, Table 1A), a greater number of markers (*P* = 0.007, Table 1B), and a higher genome coverage (*P* = 0.007, Table 1D). Average marker spacing of LM3 maps is not significantly smaller than that of JM maps (*P* = 0.099, Table 1C). Across all six, interrelated F_2_ families, coefficients of correlation (*r^2^*) between rank orders of markers on RG and ML maps are equal to or above 0.9999, as expected from the iterative method of map construction, while coefficients of correlations (*r^2^*) between rank orders of markers on RG and LM3 maps range from 0.9967 to 0.9991 (Figure 1).

**Table 1:** Student’s *t*-tests on comparing sum of lengths (A), total no. of markers (B), average spacing (C), and genome coverage (D) between linkage maps constructed using JoinMap 4.1 and Lep-MAP3

	(A) Sum of lengths		(B) Total no. of markers		(C) Average spacing (cM)		(D) Genome coverage	
Family	JoinMap 4.1	Lep-MAP3	JoinMap 4.1	Lep-MAP3	JoinMap 4.1	Lep-MAP3	JoinMap 4.1	Lep-MAP3
23×31	454.6	943.0	1,032	1,660	0.445	0.572	0.862	0.863
23×40	466.3	826.2	636	1,504	0.745	0.553	0.860	0.863
31×23	585.8	640.9	760	999	0.781	0.648	0.861	0.862
40×92	540.7	806.7	790	1,679	0.693	0.483	0.861	0.863
47×92	497.1	791.3	665	1,119	0.759	0.714	0.861	0.862
92×40	589.7	585.8	699	885	0.856	0.67	0.861	0.862
*Mean*	522.367	765.667	763.667	1307.667	0.713	0.607	0.861	0.863
*t statistics*	−3.201		−4.392		2.02		−4.392	
*p-value*	0.024		0.007		0.099		0.007	

Most of the 67,635 SNPs, which are mapped by either JM or LM3 (Table 2), belong to the consensus linkage group for their contig (65,167 or 96.4%), are mapped by LM3 (65,133 or 96.3%), or are mapped in only a single family (49,609 or 73.3%). A three-way loglinear analysis (Table 3) finds significant two-way interactions between assignment to consensus linkage group and mapping method (χ^2^ = 14.2, 1 df, *P* = 0.0002), and between assignment to consensus linkage group and number of mapping families or level of family support (χ^2^ = 54.3, 1 df, *P* < 0.0001). JM is ∼1.72 times more likely to assign SNPs to the consensus linkage group than LM3, for SNPs mapped in only one family, and 2.56 times more likely, for SNPs mapped in more than one family (Table 4). For SNPs assigned to the consensus linkage group, those mapped by LM3 are ∼1.4 times more likely than those mapped by JM to be present in only one family, while for SNPs not assigned to the consensus linkage group, there is no association between mapping method and number of mapping families (Table 4). For both mapping methods, SNPs mapped in more than one family are more likely to be correctly assigned to the consensus linkage group than SNPs mapped in only a single family (odds ratios of 4.63 and 3.12 for JM and LM3, respectively; Table 4).

**Table 2:** Number of SNPs in each combination of mapping method (*i.e.*, JoinMap 4.1 *vs.* Lep-MAP3), grouping accuracy (*i.e.*, whether a SNP is assigned to the consensus linkage group for its contig, conLG, or not, non-conLG), and level of family support (*i.e.*, whether a SNP is mapped in one or more than one family)

	JoinMap 4.1		Lep-MAP3		
	1 family	>1 family	1 family	>1 family	totals
conLG	1,607	846	45,795	16,919	65,167
non-conLG	44	5	2,163	256	2,468
totals	1,651	851	47,958	17,175	67,635

**Table 3:** Analysis of maximum likelihood parameter estimates from the three-way loglinear model. Sources, as defined in caption to Table 2

Source	d.f.	Chi-square	*P*
grouping accuracy	1	12,198.3	<0.0001
mapping method	1	4,407.8	<0.0001
level of family support	1	290.8	<0.0001
grouping accuracy × mapping method	1	14.2	0.0002
grouping accuracy × level of family support	1	54.3	<0.0001
mapping method × level of family support	1	0.4	0.529
grouping accuracy × mapping method × level of family support	1	0.8	0.386

**Table 4:** 2×2 contingency tables, testing whether grouping accuracy, mapping method, and level of family support (see Table 2) are independent within each layer of the three factors. The odds ratio is the product of the upper left cell and the lower right cell divided by the product of the upper right cell and the lower left cell

Factor 1, level 1	Factor 2		Statistics		Factor 1, level 2	Factor 2		Statistics
	Level 1	Level 2				Level 1	Level 2	
***1 family***	non-conLG	conLG	*p-value*:	0.0003	*>1 family*	non-conLG	conLG	*p-value*:	0.0314
Lep-MAP3	2163	45795	d.f.:	1	Lep-MAP3	256	16919	d.f.:	1
JoinMap 4.1	44	1607	odds ratio:	1.725	JoinMap 4.1	5	846	odds ratio:	2.560
***conLG***	JoinMap 4.1	Lep-MAP3	*p-value*:	2.52E-16	***non-conLG***	JoinMap 4.1	Lep-MAP3	*p-value*:	0.9320
>1 family	846	16919	d.f.:	1	>1 family	5	256	d.f.:	1
1 family	1607	45795	odds ratio:	1.425	1 family	44	2163	odds ratio:	0.960
***JoinMap 4.1***	1 family	>1 family	*p-value*:	0.0004	***Lep-MAP3***	1 family	>1 family	*p-value*:	4.22E-72
non-conLG	44	5	d.f.:	1	non-conLG	2163	256	d.f.:	1
conLG	1607	846	odds ratio:	4.633	conLG	45795	16919	odds ratio:	3.122

### A compendium of mapped SNPs shows evidence for chimeric contigs

We constructed a compendium of 71,156 SNPs, which were placed on at least one of 19 linkage maps for 12 mapping families and were successfully remapped from the v9 genome assembly to the Chr_v1 assembly (Table S5). Using a decision tree (Figure 2), we classified 68,851 of these SNPs as having been mapped with high-confidence. Not assigned to the consensus linkage group for their contig were 2,524 SNPs (3.6%), of which 962 were single exceptions to surrounding SNPs mapping to the consensus linkage group, 1,364 were in blocks of contiguous SNPs assigned to a non-consensus linkage group but in only one family, and the remaining 198 were in blocks of contiguous SNPs mapped to a non-consensus linkage group in more than one family. This last group suggests that 74 contigs (of 275 contigs containing high-confidence SNPs) have minor assembly problems, consisting of blocks of 3.81 SNPs, on average, with a median length of 33 base pairs (bp), which map to a linkage group different than the consensus linkage group for the containing contig (Table S6). The total length of these “chimeric” blocks, calculated as the sum of lengths of all chimeric blocks (Table S6) is 446,528 bp.

### A linkage-based assembly of the C. gigas genome

ALLMAPS successfully places all high-confidence SNPs on 19 linkage maps from 12 families (Table S7A). Anchoring 288 contigs with these high-confidence SNPs, ALLMAPS assembles a chromosome-level *C. gigas* genome of 587,333,624 bases, accounting for 98.9% of the Chr_v1 genome assembly (Table S7B). Adding a default gap of 100 bases between contigs, ALLMAPS generates a genome totaling 587,361,424 bases, with chromosomes ranging from 39,883,917 to 78,822,797 bases in length (Table S7C). The remaining contigs, the total length of which account for only 1.1% of the Chr_v1 assembly, do not contain any high-confidence SNPs and, thus, are not placed by ALLMAPS. Comparing the sequence-based and linkage mapping-based assemblies, contig-by-contig, we find that the 275 contigs in common to both assemblies fall into five categories (Table S8): (1) 205 contigs (74.5%) assigned to the same chromosome and assembled in the same order by genetic and sequencing methods (*i.e.*, identical contigs); (2) 26 contigs (9.4%) assigned to the same chromosome but in different orders between the two assemblies; (3) 31 contigs (11.3%) assigned to the same chromosome, assembled in the same order, but reversed in orientation between the two assemblies; (4) nine contigs (3.3%) whose orientation in the ALLMAPS assembly is unknown; and (5) four contigs (1.4%) assigned to different chromosomes in the two assemblies. The 205 contigs that are assembled the same way by both assemblies, plus the 40 contigs assembled consistently but with reverse or unclear orientation, account for 89.1% of the 275 contigs in common (Table S8B). Of the 26 contigs that are placed in different orders, 18 are within three positions of aligning.

### Recombination rates for six, interrelated F_2_ families

**Figure 3:**
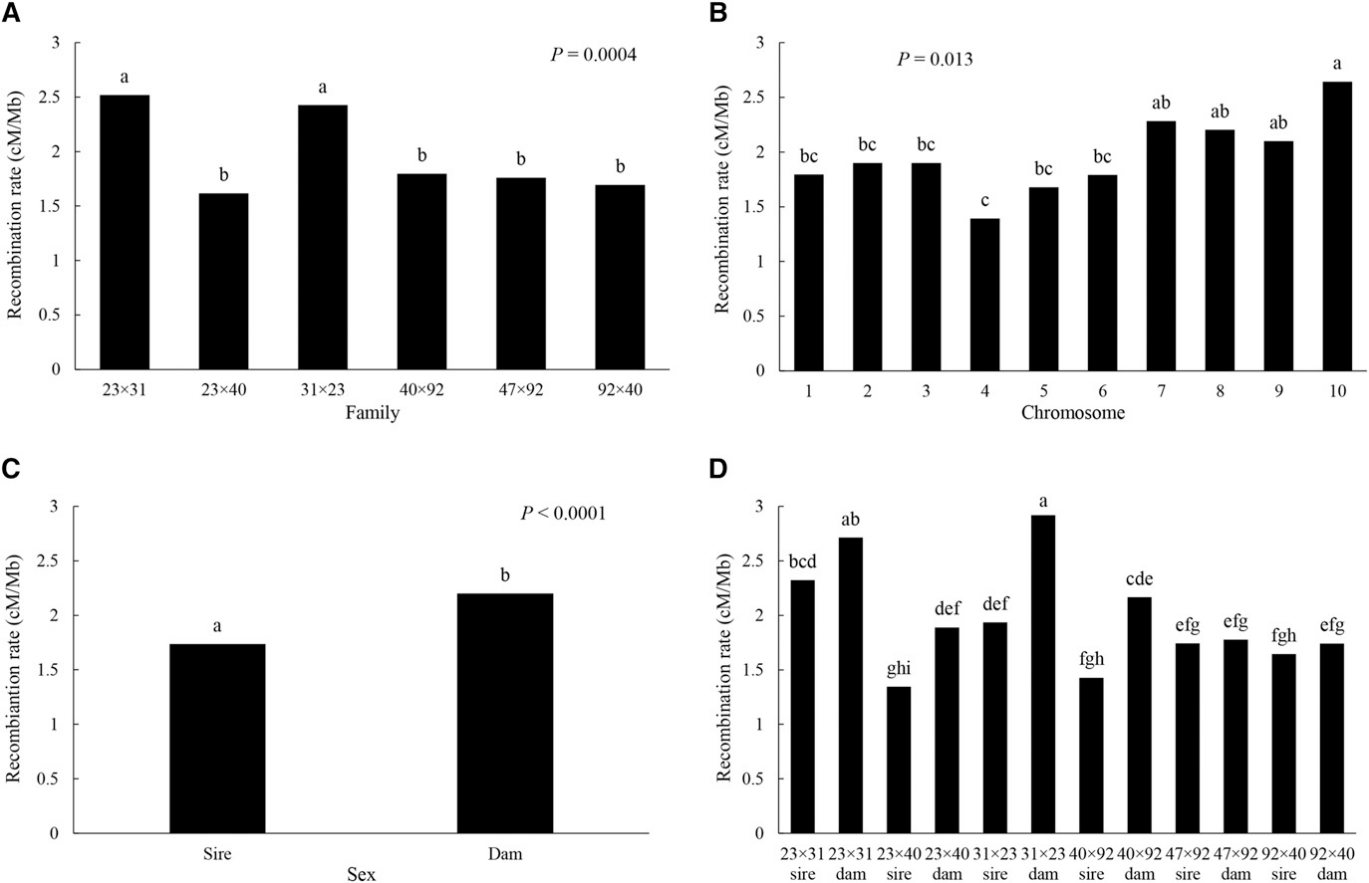
Recombination rate (cM/Mb) by family (A), chromosome (B), sex (C), and parent (*i.e.*, family × sex) (D).

Mean recombination rates (RR), across 10 chromosomes and the 12 parents of the six, interrelated F_2_ families, were analyzed by three-way ANOVA, with family, parent, and chromosome as the factors. The model is highly significant (*F*_74/45_ = 2.86, *P* = 0.0001, *r*^2^ = 0.824), yielding a grand mean RR = 1.97 cM/Mb (Table S9). As the two-way interaction of family × chromosome is significant (*P* = 0.032), we test the significance of family and chromosome with this interaction term, finding that both main factors are significant (family, *P* = 0.0004; chromosome, *P* = 0.013; Table S9). Neither of the two-way interactions involving sex is significant, though the one with family is marginal (*P* = 0.075); the main factor, sex, is highly significant when tested with the error term (*P* < 0.0001; Table S9). Two families, 23×31 and its reciprocal 31×23, have significantly higher RR (2.52, 2.43, respectively) than all other families (average RR = 1.72), which are not statistically different (Figure 3A). Chromosomes 4 and 10 represent the extremes of chromosome-wide RR (1.39, 2.64, respectively), with no significant difference among the remaining chromosomes (Figure 3B). Dams have significantly higher recombination rates than sires (Figure 3C; 2.20 *vs.* 1.74). Finally, a plot of the marginally significant family × sex interaction shows that dams consistently have higher, though not necessarily significantly higher RR than sires across families and that the dams for the reciprocal 23×31 and 31×23 families appear to account for the high recombination rates of those families (2.71, 2.92, respectively; Figure 3D).

**Figure 4:**
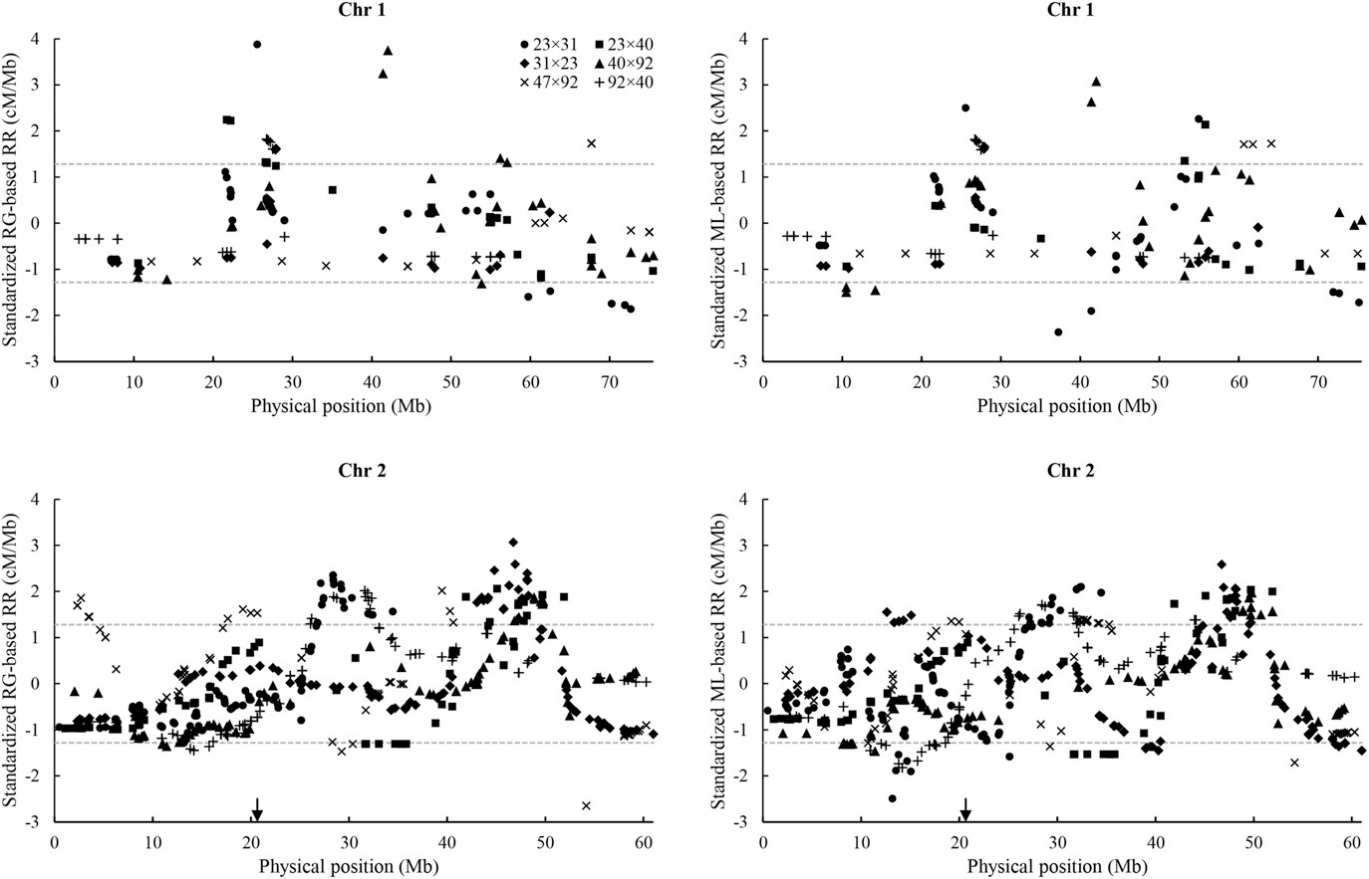

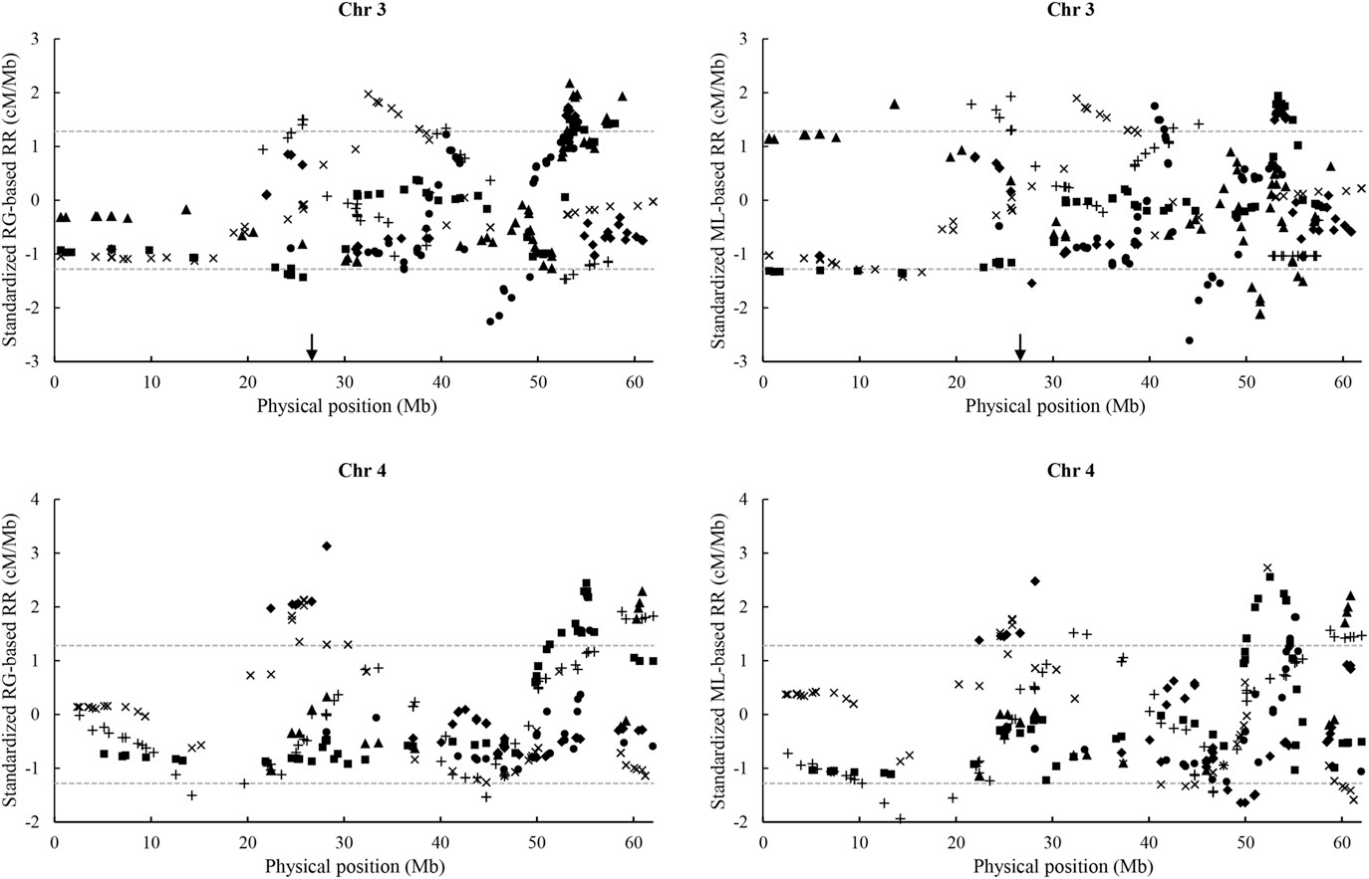

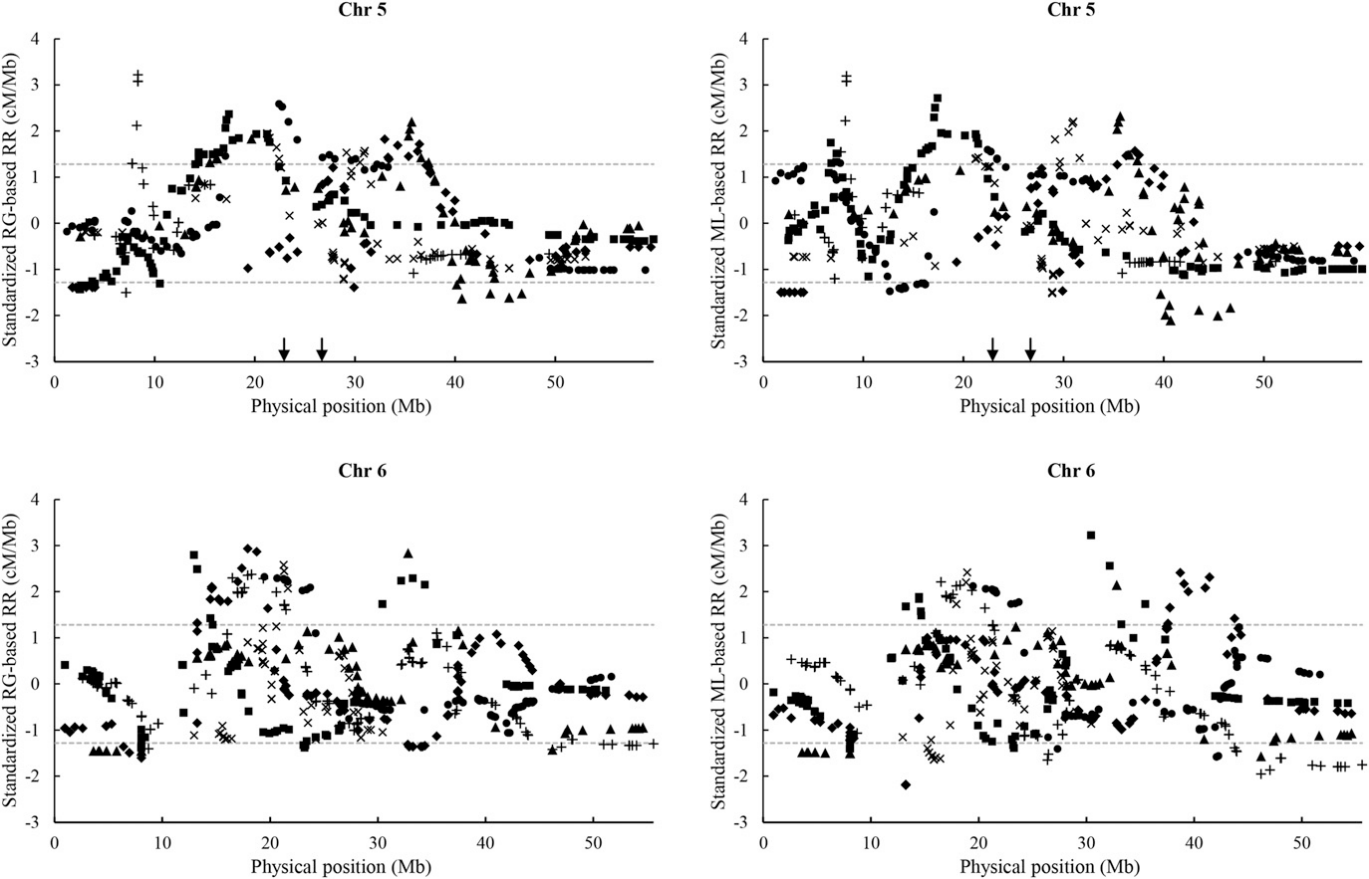

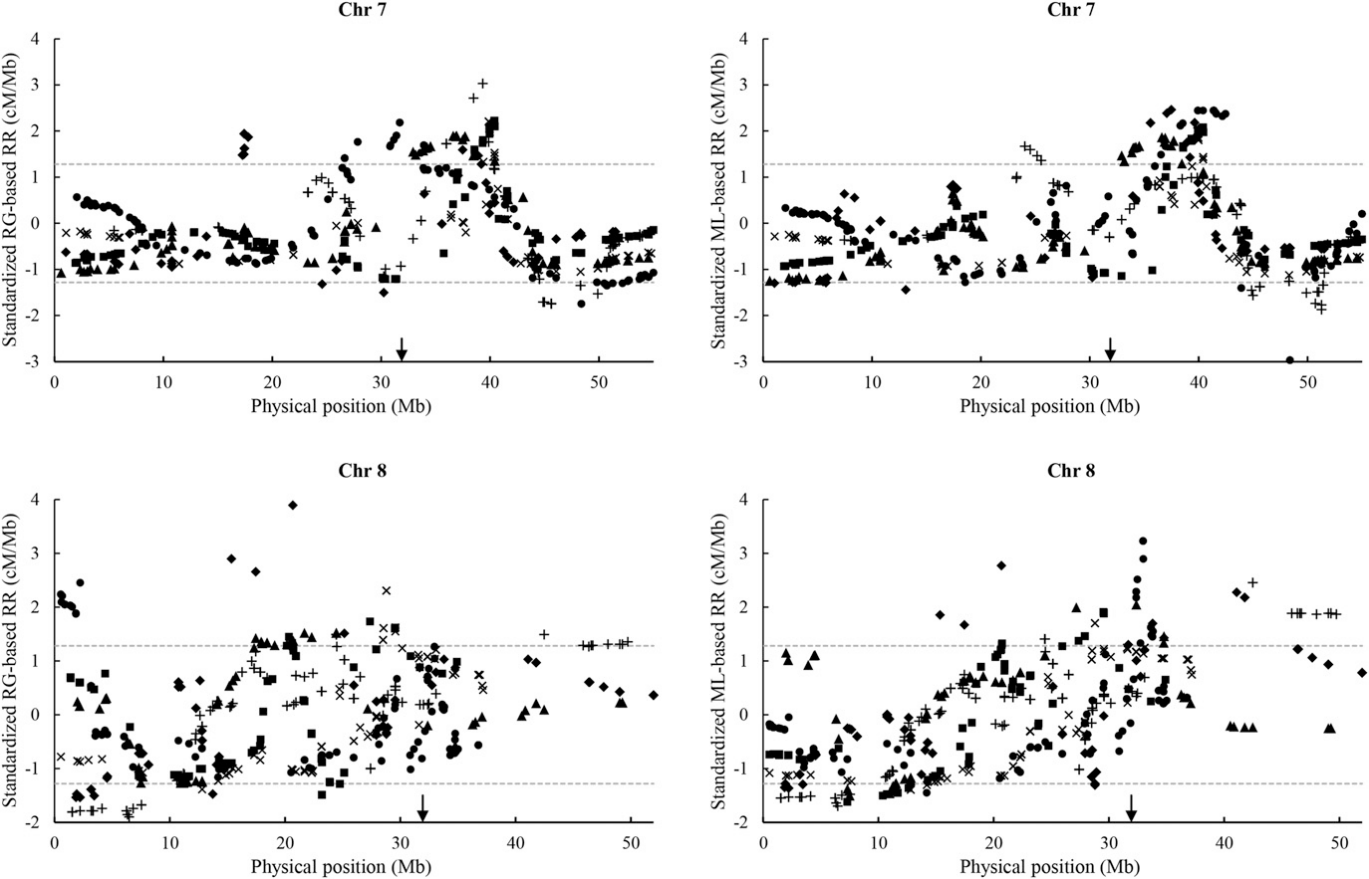

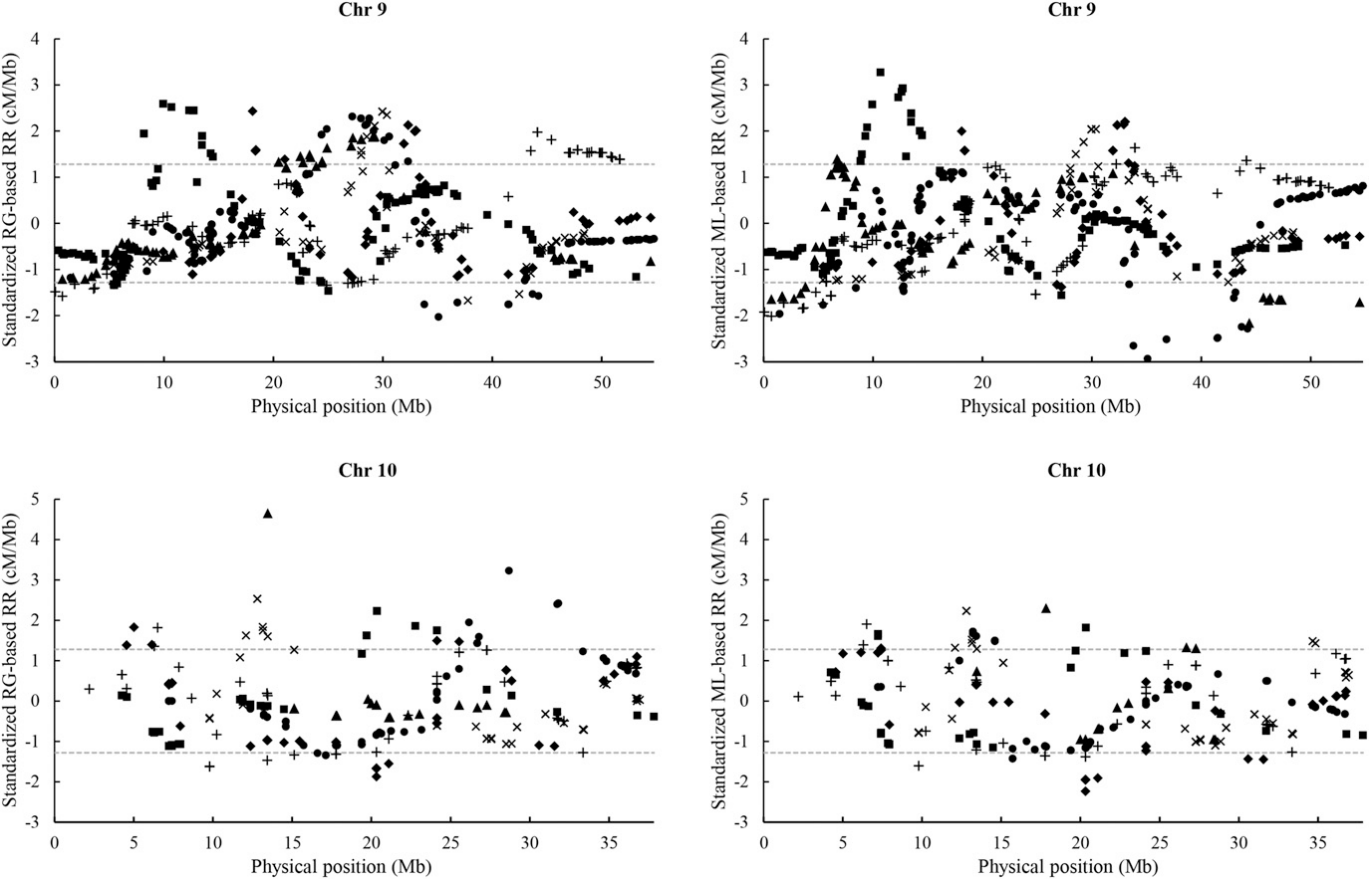
Standardized RG- and ML-based recombination rates (RR, cM/Mb) along ten chromosomes of the Chr_v1 genome assembly for six, interrelated F_2_ families. Dashed lines indicate the nominal 10^th^ percentile (-1.28) and 90^th^ percentile (1.28) of the standardized RR. Arrows indicate the leftmost position of microsatellite markers linked to centromeres (Hubert *et al.* 2009).

In addition to differences in recombination among families and chromosomes and between the sexes, we also observe variation in recombination rate along each chromosome (Figure 4). Random distribution of standardized RR values is rejected for 17 of 20 tests of values below -1.28 and for 26 of 30 tests of values above 1.28 (Table S10), suggesting that recombination hotspots and coldspots exist on all chromosomes but the first and in all families. For the five chromosomes, to which we could confidently map microsatellite DNA markers tightly linked to the centromere (Hubert *et al.* 2009; chromosomes 2, 3, 5, 7, and 8), however, we found only a random distribution of centromere-associated RR values across families and, thus, no evidence for reduced recombination around centromeres.

## Discussion

### Linkage mapping by GBS

With the advent of GBS methods, the generation of a large number of markers for linkage mapping has become cost-efficient. In this study, we generated from 4,615 to 13,885 useful SNPs for 12 mapping families with GBS. Incorporating this number of markers into linkage maps is still challenging. We used two different linkage-mapping strategies, JoinMap (JM; Van Ooijen 2011), which requires pre-processing of sequence data to call genotypes but allows for iterative mapping procedures, using regression (RG) and maximum likelihood (ML) methods, and Lep-MAP3 (LM3; Rastas 2017), which calculates genotype likelihoods from variant call format (vcf) files or binary alignment (bam) files and proceeds directly to determination of linkage. By comparing JM’s RG and ML maps and LM3 maps for the six, interrelated F_2_ families, we find all three maps are consistent with each other (Figure 1), suggesting that the ordering of markers by JM and LM3 is consistent.

Next, we considered the mapping of a given SNP to the consensus linkage group of SNPs on the contig containing that SNP as a correct mapping. Loglinear analysis of the mapping data shows that JM correctly maps markers more frequently than LM3 does, when a single mapping family is available but especially when multiple mapping families are available (Tables 2-4). Thus, JM, especially when its RG and ML methods are coupled in an iterative manner to cross-check marker orders, produces more reliable maps than LM3, but at a substantially greater cost in time and effort. This tradeoff was worthwhile, to produce accurate maps in the early stages of linkage analysis for the Pacific oyster, which has a substantial load of deleterious mutations causing segregation distortion (Launey and Hedgecock 2001; Hedgecock *et al.* 2015). For species in an early stage of linkage analysis that have less segregation distortion and, now, for the oyster, with the convergence of linkage and physical maps, as demonstrated here, the efficiency and automation of LM3 becomes an attractive option for map construction. For either mapping method, SNPs mapped in more than one family are more likely to be correctly mapped than SNPs mapped in only a single family. Thus, as found previously (Hedgecock *et al.* 2015), use of multiple families, especially those related by descent and sharing markers, lends confidence in the statistical construction of linkage maps.

GBS generates a large number of markers, but a large number of markers will not necessarily increase map densities, especially if most markers are mapped to identical positions, because the number of recombination events is limited by the size of the mapping family. In our study, LM3 maps have 13 to 22 times more markers than RG maps, but over 90% of markers on LM3 maps are assigned to identical positions, compared to an average of only 30% for RG maps. Therefore, improving the resolution of linkage mapping and subsequent studies, such as QTL mapping, requires increases in sample sizes rather than increases in the number of markers.

### Assessment of the Chr_v1 *C. gigas* genome assembly at the contig level

Seventy-four of 275, Chr_v1 contigs (26%) with high-confidence SNPs contain blocks of two or more SNPs mapped in two or more families to a linkage group different than the consensus linkage group for their contig (Table S6). The total length of these “chimeric” blocks, however, amounts to only 0.08% of the total length of the assembly (Table S6, Table S7), and the chimeric blocks themselves are small, with a median length of only 33 bp (Table S6, Figure S1). The lower proportion of chimeric contigs and the much smaller size of chimeric blocks suggest that the Chr_v1 assembly of the *C. gigas* genome is a substantial improvement over the v9 genome assembly (Zhang *et al.* 2012), with a high proportion of its largest scaffolds composed of large blocks mapping to different linkage groups (Hedgecock *et al.* 2015). As observed for chimeric scaffolds in the v9 genome assembly, chimeric contigs tend to be longer than non-chimeric contigs, suggesting that assembly errors are more likely to occur in longer than in shorter contigs.

### Assessment of the Chr_v1 genome assembly at the chromosome level

We assembled contigs into chromosomes, using ALLMAPS, and compared the order and orientation of 275 contigs in common with the Chr_v1 assembly. Overall, nearly 90% of these contigs are identically assembled by HiC analysis and ALLMAPS. Inconsistencies between the two assemblies for the remaining 10% of contigs may be largely driven by an insufficient number of high-confidence SNPs for accurate assembly based on linkage information. Identically assembled contigs contain 12 to 1,788, high-confidence SNPs, with a mean of 317 (Table S8B), while contigs with reversed or unclear orientation contain 1 to 403 SNPs, with a mean of 72. In contrast, 26 contigs that are ordered differently on the two assemblies contain 2 to 119 SNPs, with a mean of 36 (Table S8B); nevertheless, the median difference in order of these contigs is less than three places.

The four contigs (1.4%) assigned to different chromosomes by HiC analysis and ALLMAPS contain only three to 14, high-confidence SNPs (Table S8B). Contig 237, which contains 14, high-confidence SNPs, contains 26 SNPs in total, 14 of which are assigned to LG2, while 12 are assigned to LG10; LG10 is consistent with the chromosome assignment by HiC analysis (Table S5, Table S8A). While the 12 SNPs assigned to LG10 are excluded from ALLMAPS, because they do not pass the filtering criteria for high-confidence SNPs, we still suspect that contig 237 could either be part of chromosome 10 of the ALLMAPS-generated assembly or could comprise two pieces that should be assigned to chromosomes 2 and 10. Both possibilities may suggest a potential contig misassembly, but we cannot draw a firm conclusion with limited information at this point. For the remaining contigs (257, 298, and 315), we do not find any solid evidence for these contigs being assigned to other linkage groups, but the small number of high-confidence SNPs on them may not provide sufficient information for ALLMAPS to generate a correct assembly (Table S8A).

Altogether, Chr_v1 appears to be a reliable chromosome-level assembly of the Pacific oyster genome, which will make it invaluable for future studies. The assembly may still contain some small errors in assembly of contigs. Also, we note that we did not have enough high-confidence SNPs for checking ∼10% of contigs (*i.e.*, 30 out of 301 contigs). Linkage maps of larger families and more markers may be more helpful in assessing, and potentially enhancing this genome assembly.

### Variation in recombination rate

Our compilation of linkage maps for the Pacific oyster, in combination with a chromosome-level sequence assembly affords an opportunity, for the first time, to profile recombination rates (RR) across a Lophotrochozoan genome. In addition, information from a set of interrelated F_2_ families permits partitioning of variance in RR among parents and chromosomes and between the sexes to shed light on causes of variation in this fundamental process.

Recombination rate varies significantly among families, among chromosomes, and between the sexes. Reciprocal families 23×31 and 31×23 have similar RRs that are significantly higher than those in the other four families, suggesting, perhaps, a heritable basis for variation in recombination rate that bears further investigation. Across family and sex, chromosomes 10 and 4 have the highest and the lowest RRs, respectively; the population and evolutionary consequences of this nearly twofold difference in RR merits further exploration as well. As reported previously (Hubert and Hedgecock 2004; *cf*. Hedgecock *et al.* 2015), maternal parents have significantly higher RRs than paternal parents in the Pacific oyster, although the extent of this difference varies among families (marginally significant family × sex interaction; see Figure 3D, Table S9). That such a difference exists between sibling F_1_ hybrids, in a species with non-chromosomal and labile sex determination (Hedrick and Hedgecock 2010), suggests that sex-specific recombination is determined by sex-specific differences in physiology or gametogenesis rather than genetic factors. It would be interesting to determine, as is possible with sex-reversing oysters, RRs for the same individual as a male and a female parent.

Although we expect to see lower recombination around centromeres (Stapley *et al.* 2017), which should be located in the medial regions of the oyster’s metacentric or sub-metacentric chromosomes (Thiriot-Quievreux 1984), such a pattern is not evident in the recombination rate profiles (Figure 4). A more detailed analysis was made possible by locating putative centromeres on five chromosomes, using microsatellite markers tightly linked to centromeres in half-tetrad analyses (Hubert *et al.* 2009). Minimum recombination rates in these putative centromere-containing regions appear to be a random sample of genomic recombination rates, however, so we have no evidence for reduced recombination in the vicinity of the centromeres. As half-tetrad analyses are quite tractable in the oyster, it should be possible to map centromeres more precisely in the future.

Finally, we find suggestive evidence for recombination hotspots and coldspots on all chromosomes but the first and across all six, interrelated F_2_ families (Figure 4). Some hotspots and coldspots appear to be shared across two or more families (*e.g.*, hotspots at ∼49 Mb on chromosome 2, in 23×40, 31×23, and 40×92, ∼53 Mb on chromosome 3, in 23×40 and 31×23, and ∼40 Mb on chromosome 7, in 23×40, 40×92, and 47×92; a coldspot at ∼46 Mb on chromosome 6 in the reciprocal F_2_ hybrids 40×92 and 92×40; Figure 4).
